# Assessment of electrolytes, markers of glycaemic control and renal dysfunction among adult Nigerians recently diagnosed with type 2 diabetes mellitus

**DOI:** 10.4314/ahs.v22i3.31

**Published:** 2022-09

**Authors:** Oloruntoba A Ekun, Oloruntoba F Fagbemi, Esther N Adejumo, Oyeronke O Ekun, Kehinde S Wojuade, Folu M Oshundun, Florence O Adefolaju, Sade R Oyegbami

**Affiliations:** 1 Department of Medical Laboratory Science, College of Medicine University of Lagos; 2 School of Medical Laboratory Science, Lagos University Teaching Hospital; 3 Department of Medical Laboratory science Babcock University Ilishan Remo, Ogun State; 4 Department of Haematology and Blood group Serology, College of Medicine University of Lagos

**Keywords:** Type 2 diabetes mellitus, renal disease, glycated haemoglobin, high sensitivity C-reactive protein

## Abstract

**Background:**

Diabetes mellitus is a chronic and progressive endocrine disorder that may result in macro and microvascular complications.

**Objective:**

This study assessed some biochemical analytes in Nigerians who were recently (≤ 6 months) diagnosed with Type 2 diabetes mellitus (T2DM).

**Methods:**

160 T2DM and 90 non-diabetic control participated in this study. Blood samples were collected and analyzed for Heart-type fatty acid-binding protein (HFABP), high sensitivity C-reactive protein (hs-CRP), electrolytes, lipid and renal profile parameters, glycated haemoglobin (HBA1C) and fasting blood glucose (FBG), using standard guidelines.

**Result:**

The body mass index (BMI) of the T2DM volunteers was higher than control (P <0.001). The lipid profile, potassium, glucose, HBA1C, urea and creatinine values were elevated (P <0.001) while estimated glomerular filtration rate (eGFR) was lower (P<0.05) in diabetes. The median HFABP and hs-CRP were raised (P <0.05) in T2DM. Positive associations existed between FBG and urea (P <0.001), Creatinine and HBAIC (P <0.001). A logistic regression analysis, shows that an increased BMI, HBA1C, FBG, Cholesterol, urea and creatinine were associated with higher odds (p<0.001) of cardiovascular and renal complications.

**Conclusion:**

Elevated hs-CRP, glycated haemoglobin, urea and creatinine among T2DM increase the odds of cardiovascular and renal insults in this population.

## Introduction

Diabetes mellitus (DM) is one of the largest global health emergencies of the 21^st^ century^1^. There are about 415 million people living with diabetes mellitus worldwide, with type 2 diabetes (T2DM) accounting for more than 90% of diabetic patients^2^. Diabetes mellitus is a major risk factor for cardiovascular disease (CVD), which is the most common cause of death among adults with DM^3^. Besides the well-recognized microvascular complications of DM, such as nephropathy and retinopathy, there is a growing epidemic of macrovascular complications, including diseases of coronary arteries, peripheral arteries, and carotid vessels, particularly in the burgeoning type 2 DM populations^4^.

DM is a group of metabolic diseases marked by high levels of blood glucose resulting from problems in insulin production, insulin use, or both. The data from the International Diabetes Federation indicated that an estimated 415 million adults aged 20–79 years worldwide have DM in 2015 and the number will project to 642 million in 2040, with the prevalence increasing from 8.8 to 10.4%. Despite the high prevalence of diagnosed DM, as many as 193 million people representing close to half of all people with DM are unaware of their disease. The prevalence of Diabetes Mellitus continues to increase. The current prevalence of diabetes mellitus in Nigeria is between 5–6%^5^ with a current African Region prevalence of between 2.1–6.7%^6^. Regionally, the age-adjusted prevalence of DM is 7.3% in Europe, 10.7% in the Middle East and North Africa, 11.5% in North America and Caribbean, 9.6% in South and Central America, 9.1% in Southeast Asia, and 8.8% in Western Pacific. China, India, and the USA remain the top three countries with the largest number of people with Diabetes mellitus^4^.

The two main types of DM are type 1 DM, type 2 DM. Type 1 DM is one of the most common chronic autoimmune disorders that typically manifests in early childhood and adolescence^7^. Gestational DM is a form of glucose intolerance diagnosed during the second or third trimester of pregnancy. Type 2 DM is the most common type and accounts for about 90–95% of all diagnosed cases of DM. The number of people with type 2 DM is growing rapidly worldwide. This rise is associated with aging population, economic development, increasing urbanization, less healthy diets, and reduced physical activity^1^. Many people remain undiagnosed because there are often few symptoms during the early years of type 2 DM or symptoms that do occur may not be recognized as being related to DM. However, during this time the body is already being damaged by excess blood glucose, and as a result, many people are affected by complications even before diagnosed with type 2 DM.

Consistently high blood glucose levels can lead to serious diseases associated with heart, blood vessels, eyes, kidneys, and nerves. The cardiovascular diseases (CVDs) that accompany DM include angina pectoris, myocardial infarction, stroke, peripheral artery disease (PAD), and congestive heart failure (CHD)^8^,^9^. It has been estimated that about 53% of life time medical cost of managing type 2 diabetes mellitus is dedicated to managing some of these complications in the USA^10^; however there is a paucity of such data in many developing countries especially in Nigeria, thus prevention of complications through rigorous glycaemic monitoring may be invaluable.

It has been reported previously that diabetes mellitus is an independent risk factor for cardiovascular diseases (CVD)^8^. The adverse influence of diabetes extends to all components of the cardiovascular system: the microvasculature, the larger arteries, and the heart, as well as the kidneys^11^. Patients with diabetes mellitus aggregate other comorbidities such as obesity, hypertension, and dyslipidemia which also contribute to increase the risk for CVD. This study aims to assess Heart-type fatty acid-binding protein (HFABP) (cardiac biomarker), markers of glycaemic control and renal function among individuals recently diagnosed with type 2 diabetes mellitus.

## Materials And Methods

### Study design

This is a cross-sectional study on recently diagnosed type 2 Diabetes Mellitus (within first six months of diagnosis) attending Diabetic Clinic of a General Hospital, Lagos State. A total number of 250 volunteers participated in this study out of which 160 were recently diagnosed with diabetes mellitus. The remaining 90 volunteers were non-diabetic participants. This group served as control.

### Human Subjects

Adults (≥40 years) diagnosed of diabetes mellitus. Studies have shown that type 2 Diabetes Mellitus is common among men and women older than 40years. However recent study shows that type 2 diabetes mellitus is becoming common among Children and adolescents^5^. American Diabetes Association (ADA)^12^, has also reported that the prevalence by age and sex appeared more between ages 20 – 79 years with a peak age of 50 - 59 years.

### Sample size

The calculation of the sample size for this study was based on the prevalence of between 5–6% for this disorder in Nigeria based on literature^5^.

### Criteria for selection

#### Inclusion criteria

Adult male and female ≥40 year's old consented patients who were recently registered (not later than 6 months) at the Diabetic Clinic and who do not have any other comorbidity as at the time of this study were recruited.

#### Exclusion criteria

Children and teenager were excluded from this study, also pregnant women as well as individuals with any form of malignancies were excluded from this study. Diabetes subjects with history of smoking and malaria parasite infestation or have been treated for malaria in the past one month before this study were excluded from this study. Also this study excluded diabetes mellitus individual with overt evidence of co-morbidity (renal failure and hypertension) as at the time of this study.

#### Medication for diabetes volunteers:

Majority of the diabetes participants (Diabetes) were mainly on combination of two of the following; Metformin-a Biguanides, Diamicron MR (Gliclazide)- a Sulfonylureas and Voglinorm (Voglibose)-an α-Glucosidase inhibitors.

### Ethical approval and Informed consent

Approval for this study was obtained from the Health Research Ethics Committee (HREC) (approval number: CMUL/HREC/04/19/516) of College of Medicine of the University of Lagos prior to the commencement of the study. Informed consent was obtained from each participant before the commencement of this study after the purpose and the objectives of the study have been explained to them. This study was in total conformity to the declaration of Helsinki.

### Blood sample collection and handling

After 8–12 hours overnight fast, a total of ten (10) mls of venous blood was collected and dispensed into plain, lithium heparinized, K2EDTA and fluoride oxalate bottles. All the bottles except K2EDTA samples were centrifuged at 5,000 rpm for three (3) minutes to separate serum and plasma respectively. The serum and plasma were extracted into Eppendorf tubes and stored at -200C until the day of analysis.

## Methods

Electrolytes were determined using Ion Selective Electrode (ISE), while lipid, urea, creatinine, glucose and glycated haemoglobin (HBA1C) were analyzed using Roche-Cobas 111. High Sensitivity C Reactive Protein (hs-CRP) and Heart Type Fatty Acid Binding Protein (H- FABP) were determined using ELISA method. Estimated glomerular filtration rate (eGFR) was calculated using Modification of Diet in Renal Disease (MDRD) equation^13^.

### Data analysis

The data were analyzed with Software for Statistics and Data Science [STATA software version 16 (StataCorp) USA]. The mean of age, body mass index, systolic and diastolic blood pressure were compared between type 2 diabetes mellitus and non-diabetic volunteers. Test of normality was conducted on all continuous variable using kurtosis, Skewness, Shapiro wilk and Kolmogrorov-Simrnov test. Variables that were normally distributed were analyzed using parametric test while the skewed continuous variables were analyzed using non-parametric method. Normally distributed variables were presented as mean standard deviation while the skewed variables were presented as median and interquartile ranges. A normally distributed independent continuous variables were analyzed using independent student t test while a non-normally distributed variables were analyzed using Wilcoxon Mann-Whitney U test. The degree of association of the measured parameters were determined using Pearson Correlation. Spearman Rank Correlation was used to analyze the skewed variables. A multivariate logistic regression analysis was conducted to predict the risks of renal and cardiovascular derangement among the study participants. The level of statistical significance was set at probability of less than 0.05 (p<0.05).

## Results

The results of the present study are shown in Table 1 to 5. Table 1 presents the Anthropometric parameters and blood pressure of the participants. The mean age (years) of the test and control groups was 59±8.97 and 60±10.97 respectively. The mean weight (Kg) and BMI (Kg/m2) of the participants were: 79.59±9.83; 70.62±4.73; 31.76±4.32 and 27.94±3.39 for test and control group respectively. Systolic and diastolic blood pressure (mmHg) measurements were 141.09±10.98; 129.07±4.21, 88.71±9.21 and 77.81±6.67 for type-2 diabetes mellitus and control groups respectively. The electrolytes and renal function parameters were estimated for test and control groups.

**Table 1:** Anthropometric parameters and blood pressure of the Participants

Variables	Test (mean±SD) n(160)	Control (mean±SD) n(90)	t value	p value
Sex	Female: 102(63.75%) Male: 58(36.25%)	Female: 38(42.12%) Male: 52(57.78%)		
Age (year)	59±8.97	60±10.97	0.47	0.64
Height (m)	1.58±0.07	1.60±0.07	1.28	0.20
Weight (Kg)	79.59±9.83	70.62±4.73	8.14	<0.001*
BMI (Kg/m^2^)	31.76±4.32	27.94±3.39	7.39	<0.001*
Systolic Bp (mmHg)	141.09±10.98	129.07±4.21	9.97	<0.001*
Diastolic Bp (mmHg)	88.71±9.21	77.81±6.67	9.87	<0.001*
History of smoking	No	No		
History of recent malaria infestation/medication	No	No		

* Level of significance is p< 0.05

**Table 2:** Comparative analysis of the Electrolytes, Renal function and lipid profile parameters of the Participants

Variables	Test (mean±SD)	Control (mean±SD)	p value
	n(160)	n(90)	
Sodium (mmol/l)	139.89±2.79	139.29±3.45	0.137
Potassium (mmol/l)	4.56±0.55	3.81±0.24	<0.001*
Chloride (mmol/l)	104.64±4.50	105.17±4.93	0.379
Bicarbonate (mmol/l)	22.12±2.12	21.47±2.03	0.019*
Urea (mg/dl)	32.45±9.60	26.06±5.76	<0.001*
Creatinine (mg/dl)	1.06±0.34	0.89±0.45	<0.001*
eGFR median (mL/min/1.73)	75.466	122.375	<0.001*
(Interquartile range)	(61.303–88.381)	(98.157–143.532)	
Cholesterol (mg/dl)	190.44±35.59	167.09±17.22	<0.001*
Triglyceride (mg/dl)	105.69±31.52	79.52±12.02	<0.001*
High Density	41.65±8.53	34.79±5.16	<0.001*
Lipoprotein (mg/dl)			
Low Density Lipoprotein (mg/dl)	127.73±25.22	109.59±10.85	<0.001*

* Level of significance p < 0.05

**Table 3:** Comparative evaluation of fasting blood glucose, glycated haemoglobin, Heart type fatty acid binding protein and high sensitivity-C-reactive protein

Variables	Test (mean±SD)	Control (mean±SD)	p value
	n(160)	n(90)	
FBG (mg/dl)	163.16±51.28	102.66±10.49	<0.001*
Glycated HB (HBAIC)	8.79±2.75	5.99±0.41	<0.001*
(%)			
HFABP(ng/ml) (Median)	4.56	3.93	<0.001*
(Interquartile range)	(2.58–6.92)	(1.19–5.83)	
hsCRP(mg/L) (Median)	2.20	2.05	0.038*
(Interquartile range)	(1.3–4.2)	(0.7–4.5)	

* Level of significance is p < 0.05

**Table 4:** Levels of Association between parameter measured in type 2 Diabetes Mellitus Volunteers

Variables	Correlation Coefficient “r”	p value
BMI (kg/m^2^) VS Systolic BP (mmHg)	0.1628	0.0397*
Weight (kg) VS Diastolic BP (mmHg)	0.2290	0.0035*
FBS (mg/dl) VS Systolic BP (mmHg)	0.5096	<0.001*
FBS (mg/dl) VS Diastolic BP (mmHg)	0.4988	< 0.00I*
FBS (mg/dl) VS Urea (mg/dl)	0.2948	<0.001*
Cholesterol (mg/dl) VS BMI (kg/m2)	0.2712	<0.001*
Cholesterol (mg/dl) VS Weight (kg)	0.2911	<0.001*
Systolic BP (mmHg) VS Cholesterol (mg/dl)	0.2042	0.0096*
Diastolic BP (mmHg) VS Cholesterol (mg/dl)	0.1696	0.0320*
Systolic BP (mmHg) VS Triglyceride (mg/dl)	0.2327	0.0031*
Diastolic BP (mmHg) VS Triglyceride (mg/dl)	0.2422	0.0020*
Weight (kg) VS Triglyceride (mg/dl)	0.4011	<0.001*
BMI (kg/m2) VS Triglyceride (mg/dl)	0.3740	<0.001*
HDL (mg/dl) VS Weight (kg)	0.3491	<0.001*
HDL (mg/dl) VS BMI (kg/m2)	0.3115	<0.001*
Cholesterol (mg/dl) vs Urea (mg/dl)	0.1690	0.0326*
Creatinine (mg/dl) vs Urea (mg/dl)	0.6935	<0.001*
Urea (mg/dl) vs FBS	0.2948	<0.001*
Urea (mg/dl) vs HBA1C	0.2877	<0.001*
Creatinine (mg/dl) and HBAIC (%)	0.2633	0.0008*
Variables	r_s_	p value
hs-CRP (mg/L) and HBAIC (%)	0.3060	0.0001*
hs-CRP (mg/L) and Creatinine (mg/dl)	0.2041	0.0096*
HFABP (ng/ml) and HBAIC (%)	0.0577	0.4689
Creatinine (mg/dl) and HFABP (ng/ml)	0.0124	0.8763

* Level of significance is p < 0.05

**Table 5:** Multivariate logistic regression analysis

Variables	odds ratio	Std error	P value	(95% Confidence interval)
BMI (Kg/m^2^)	1.120	0.037	< 0.001*	1.050 – 1.195
hs-CRP (mg/L)	1.161	0.064	0.007*	1.042 – 1.293
HFABP (ng/ml)	1.020	0.026	0.430	0.970 – 1.072
FBS (mg/dl)	1.149	0.020	< 0.001*	1.110 – 1.190
HBA1C (%)	52.717	29.778	< 0.001*	17.424 – 159.499
Urea (mg/dl)	1.127	0.0253	<0.001*	1.079 – 1.178
Creatinine (mg/dl)	5.811	3.031	<0.001*	2.090 – 1.151
eGFR (ml/min/1.73) |	0.968	0.005	<0.001*	0.959 – 0.977
Cholesterol (mg/dl)	1.032	0.006	<0.001*	1.0196 – 1.044
Trig (mg/dl)	1.085	0.0125	< 0.001*	1.060 – 1.109
LDL-Cholesterol (mg/dl)	1.058	0.0107	<0.001*	1.037 – 1.079

Level of significance is P < 0.05

The (Table 2) mean sodium (mmol/l), potassium (mmol/l) chloride (mmol/l), bicarbonate (mmol/l), urea (mg/dL) and creatinine (mg/dL) were: 139.89 (mmol/l), 4.56 (mmol/l), 104.64 (mmol/l), 22.12 (mmol/l), 32.45 (mg/dL), 1.06(mg/dl) respectively for type-2 diabetes mellitus volunteers. The mean cholesterol, Triglyceride, HDL and LDL were: 190.44±35.59 and 167.09±17.22, 105.69±31.52 and 79.52±12.02, 41.65±8.53 and 34.79±5.16, 127.73±25.22 and 109.59±10.85 for diabetic group and control group respectively. A comparative evaluation of markers of cardiovascular dysfunction and glycaemic control were presented in Table 3. Plasma fasting blood glucose (FBG), Glycated Haemoglobin (HBA1C), HFABP and hs-CRP were evaluated. The Plasma HFABP and hs-CRP distribution were skewed among the participants and were presented as median and interquartile range (IQR). The mean FBG (mg/dL) and HbA1C (%) for both test and control group were: 163.16±51.28 and 102.66±10.49, 8.79±2.75 and 5.99±0.41 respectively.

Tables 4 shows Pearson Correlation Coefficient and Spearman rank correlation of the measured biomarkers among diabetes mellitus. A positive association existed between blood pressure and markers of glycaemic control and markers of renal function. High sensitivity C- reactive protein associated positively with glycated haemoglobin (HBAIC) and Creatinine. There was no association between hs-CRP and HFABP. A multivariate logistic regression analysis result was presented in table 5. The odds of future complication among test participants were higher with increase in BMI (OR: 1.120), hs-CRP (OR: 1.161), FBG (OR: 1.149), HBA1C (52.717), urea (OR: 1.127), Creatinine (OR: 5.811), Cholesterol (OR: 1.032), Triglyceride (OR: 1.085) and LDL-Cholesterol. Whereas a higher eGFR value was associated with a lower odd (0.968) of renal complication.

## Discussion

In this study, biochemical markers of glycaemic control, and renal dysfunction were measured. It was observed that in the overall, 56% of the volunteers were female. The mean age of the test and control was not significantly different from each other (Table 1). However, the mean weight and body mass index (BMI) (an indication of obesity) of the diabetic volunteers were significantly higher when compared with the control group. Previous study has opined that as BMI increases, insulin resistance also increases which results in increased blood glucose level in body. An increase in BMI as observed in test volunteers is in consonance with several previous studies. Of a particular interest were the studies by Bjorntorp^14^, Mckeigue et al.,^15^ Eckel et al.,^16^ Al-Goblan^17^, that reported the influence of obesity on type 2 diabetes risk and its association with metabolic syndrome, and cardiovascular disease. Mckeigue et al., 15 and Al-Goblan, et al.,^17^ linked Obesity to many medical, psychological, and social conditions, the most devastating of which may be type 2 diabetes.

Thus, there is a strong relationship between obesity and type 2DM. Also in Table 1, we observed that the mean blood pressure (systolic and diastolic) of the T2DM individuals was significantly higher than that of the control group. This observation agrees with previous studies that suggested that T2DM is a member of metabolic syndrome that is also referred to as syndrome X 16. Previous study has also demonstrated a strong link between T2DM and hypertension 18; this was clearly demonstrated in this study.

Furthermore, an evaluation of electrolyte and markers of renal function among the volunteers shows that T2DM volunteers demonstrated some levels of electrolytes imbalance when compared with the apparently healthy control group (Table 2). In this study it was observed that the test group presented with the plasma potassium that was significantly higher than the control group. This observation regarding plasma potassium is in consonance with some previous studies. Thus, a higher mean potassium as presented by the diabetic volunteers in this study agreed with the previous observations by Alexopoulou et al.,^19^ Kim et al.,^20^. Alexopoulou et al.,^19^ reported that hyperkalemia whenever it occurs among T2DM may be suggestive of the presence of microvascular complications of diabetes mellitus; whereas Nzerue and Jackson^21^ presented the possible mechanism and causes of hyperkalemia in T2DM. It has been suggested that patients with diabetes constitute a unique high-risk group for hyperkalemia, in that they develop defects in all aspects of potassium metabolism 22. Thus, diabetes mellitus should be considered as an independent possible cause of hyperkalemia^19^. A significantly raised urea and creatinine as well as a decrease in the estimated glomerular filtration rate (eGFR) (markers of renal function) was observed among diabetic volunteers when compared with control. This might possible suggest an underlying renal dysfunction among these set of volunteers. This observation is in concordance with the previous studies which reported an underlying renal disease among diabetes mellitus population. However, it is interesting to note that the test group had presented with a lower eGFR at the early stage of type 2 diabetes mellitus presentation. This group of individuals often develop hyporeninemic hypoaldosteronism and impaired renal excretion of potassium^23^,^24^.

Moreover, in Table 3, the plasma glucose, glycated haemoglobin, Heart-type fatty acid binding protein (HFABP) and high-sensitivity C-reactive protein (hs-CRP) were significantly raised in T2DM volunteers when compared with the control volunteers. HFABP and hs-CRP have been considered as markers of cardiovascular impairment and inflammation respectively. HFABP has been shown to be released from the injured myocardium and is detected in blood within 1 hour after the onset of ischemia. Heart type fatty acid binding protein (HFABP) has been demonstrated to be a sensitive early marker of myocardial injury. Previous study had used HFABP to demonstrate the incidence of early-period cardiac ischemia in children and adolescents with diabetic-keto acidosis (DKA)^25^. However, there is a paucity of information regarding HFABP in adult onset of diabetes mellitus. Thus, elevated HFABP in type 2 diabetes mellitus volunteers may have resulted from either lower eGFR or as an indication of cardiovascular involvement. However, in this study HFABP did not produce any significant odds of future complications among adult type 2 diabetes mellitus thus possibly limiting its predictive usefulness among adult DM. Our study also demonstrated a significant increase in the value of hs-CRP among T2DM.

Previous studies have suggested that serum hs-CRP levels are higher in T2DM patients with complications than in patients without T2DM. Also, it has been shown that T2DM is associated with a low-grade inflammation^26^. Thus, it has been reported that DM and insulin resistance are associated with the overexpression of many cytokines by adipose tissue including tumor necrosis factor-α, interleukin (IL)-1, IL-6, leptin, resistin Monocyte Chemo-attractant (MCP-1), Plasminogen Activator (PAI-1), fibrinogen and angiotensin 27. The overexpression of these cytokines contributes to increased inflammation and lipid accumulation; this might have contributed to dyslipidemia observed among T2DM in this study. It has been demonstrated that CRP impairs endothelial production of nitric oxide (NO) and prostacyclin, which are vital to vessel compliance. CRP has also been shown to increase the uptake of oxidized low-density lipoprotein (LDL) in coronary vasculature walls, which can contribute to endothelial dysfunction as well as the development of atherosclerotic plaques 28. Martin-Timon et al.,^29^ reported that increased levels of hs-CRP are related with the presence and severity of coronary artery disease (CAD) and renal impairment in individuals with T2DM.

The mean values of FBS and HbA1C in T2DM patients was significantly higher when compared with the mean value of control group. A meta-analysis of previous studies among individuals with T2DM showed that an increase in glycated haemoglobin by 1% leads to about 17–18% increase risk of CVD events 30,31. Thus Hyperglycemia in T2DM encourages the activation of oxidative stress and overproduction of mitochondrial superoxide, which trigger various metabolic pathways of glucose-mediated vascular damage 32,33. Glucose which is overtly abundant in T2DM reacts with various proteins leading to an accumulation of cross-linked proteins. This cross-linked proteins damage cells and tissues and may contribute to long-term complications in diabetes, plaque formation, and atherosclerosis^34^. Previous study supports that cardiovascular mortality is significantly increased when HbA1C levels are above 8.0% in the population with diabetes 35. It must be noted that the mean glycated haemoglobin observed in this study was 8.79% among T2DM. Also, a multivariate analysis involving glycated haemoglobin in this study produced a significant odd of complications (higher risk of cardiovascular dysfunction/complications) among T2DM group studied.

Moreover, the mean lipid profile was significantly raised in T2DM. Total Cholesterol, Triglyceride and LDL -C levels were increased in T2DM, when compared with controls. These lipid profile components presented with higher odds of future complications among diabetes participants as observed from this study. Our observations agree with the previous studies by Ejuoghanran et al.,^36^ and Srinidhi et al.,^37^. Srinidhi et al.,^37^ reported that the common lipid abnormalities associated with patients with T2DM are hypercholesterolemia and hypertriglyceridemia.

Furthermore, the levels of associations that exist among parameters measured in Type 2 Diabetes Mellitus were evaluated (Table 4). The level of Fasting Blood glucose correlates positively with urea. This observation might suggest that diabetes mellitus may precipitate renal pathology and these two together are additive risk factors for CVD 29. Also in this study, there was a strong positive correlation between weight, and Blood pressure. This observation corroborates the previous observation by Vuvor^38^ who reported that overweight and high BP have independent fatal health consequences as they carry serious risk factors for several non-communicable diseases such as type 2 diabetes, heart disease, stroke, and even death. In addition to this, a positive and significant association was observed between urea and glycated haemoglobin. Our observation regarding markers of glycaemic control and renal function agreed with the previous study by Sivasubramanian et al.,^39^. A positive and significant association was observed between Creatinine and glycated haemoglobin. This observation suggests that diabetes mellitus could provide a veritable template for renal pathology. The finding from this study agreed with the previous study by Sivasubramanian et al.,^39^. Also there were significant associations between creatinine and hs-CRP as well as HBA1C and hs-CRP. These observations also agreed with the previous studies by Shaheer et al.,^40^, and Sultania et al.,^41^. These findings suggest the possible link between inflammation and diabetes mellitus as well as pathogenesis of renal disease.

A multivariate analysis study (table 5) to predicts the risk for renal and cardiovascular involvement in type 2 diabetes mellitus using logistic regression analysis indicated that an increase in body mass index, high-sensitivity C-reactive protein (hs-CRP), fasting blood glucose (FSG), glycated haemoglobin (HBA1C), urea and Creatinine as well as total cholesterol, triglyceride and LDL-cholesterol were associated with significant odds of complications among adult diabetes volunteers.

## Conclusion

From the outcome of this study, markers of renal function and glyceamic control were elevated in type 2 diabetes mellitus. Thus, rigorous glycaemic control through effective and efficient monitoring of markers of glycaemic control, could possibly prevent or delay renal impairment. This may possibly prevent or delay the onset of micro and macro-vascular complications in type 2 Diabetes mellitus in the long run.

## Data Availability

All datasets regarding this study can be obtained from the corresponding author upon reasonable request.
